# CT-guided biopsy of lung lesions using two needles in difficult and poorly cooperative patients

**DOI:** 10.1186/s40064-015-1614-2

**Published:** 2015-12-23

**Authors:** Ji Young Ha, Kyung Nyeo Jeon, Mi Jung Park, Kyungsoo Bae, Won Sup Lee, Seung Ick Cha

**Affiliations:** Department of Radiology, Gyeongsang National University School of Medicine, Gyeongsang National University Hospital, 79 Gangnam-ro, Jinju, 660-702 South Korea; Department of Radiology, Gyeongsang National University Changwon Hospital, Changwon, South Korea; Department of Internal Medicine, Gyeongsang National University School of Medicine, Jinju, South Korea; Department of Internal Medicine, Kyungpook National University School of Medicine, Daegu, South Korea

**Keywords:** Biopsy, CT-guided, Lung, Thorax, Two needles

## Abstract

The purpose of our study was to evaluate the feasibility and safety of CT-guided percutaneous lung biopsy using two needles in difficult and poorly cooperative patients; and to examine the usefulness of the malpositioned first needle in tissue sampling with a second needle. This study included 17 consecutive patients with unsuccessful first insertion of the biopsy needle in the normal lung parenchyma and re-attempted tissue sampling through another puncture site using a second needle with the first needle retained in position until completion of the biopsy. We examined the difficult factors in biopsy that led to a failed first attempt, success rate of tissue sampling, procedure-related complications, and usefulness of the malpositioned needle. There were 1 or multiple difficult factors in all patients. In all 17 patients, core samples were successfully obtained using a second needle. Post-procedure pneumothorax and parenchymal hemorrhage occurred in 4 and 3 patients, respectively. The first needle was used as a navigational reference point for lesion localization in all patients and as an anchor restricting the mobility of the lung in patients with pneumothorax or poor breath holding capacity. CT-guided needle biopsy of the lung using a second needle without removing the first malpositioned needle is feasible and safe. During biopsy procedures in difficult or poorly cooperative patients, the malpositioned needle provides a navigational reference point or serves as an anchor to hold the movable lung.

## Background

Patients with a suspected malignant lung lesion require a diagnostic histopathology, and CT-guided biopsy is a safe and widely used method for obtaining peripheral lung tissue (Connor et al. 2000; Winokur et al. 2013). Before the procedure, radiologists carefully determine the position and needle trajectory based on the lesion location and characteristics on CT scan. However, in standard CT-guided biopsy without using real-time monitoring tools such as CT fluoroscopy, the actual process of final lesion targeting is essentially performed blindly. Thus, there are risks of failed targeting of the lesion, particularly when patients are not fully cooperative, or when the targeted lesion is small or located in a difficult to approach area of the lung. Once a needle is inserted in the normal lung parenchyma distinct from the target lesion, radiologists usually remove the malpositioned needle and re-try the procedure. However, in patients who are poorly cooperative or have difficult lesions, re-targeting the lesion is expected to be unsuccessful because of patients’ poor respiration holding or pneumothorax developed after the first trial. In the selected patients, instead of removing or repositioning, we sought to target the lesion using a second needle leaving the malpositioned first needle in position.

The purpose of this study was to present our experience with CT-guided lung biopsy performed using two needles and to evaluate the feasibility and safety of the procedure. The role of the malpositioned needle, located in the normal lung parenchyma during the entire biopsy procedure, was also reviewed.

## Methods

### Patients

Our institutional research ethics board approved this retrospective study. Between March 2010 and July 2012, 17 consecutive patients with attempted tissue sampling using two needles in one biopsy session were included in the study. In these 17 patients, the first needle was inserted into the normal lung parenchyma, and the operator tried to target the lesion using a second needle while the first needle remained in position.

One radiologist who performed the biopsy procedure reviewed the biopsy records, medical records, CT-guided biopsy images, and all diagnostic images. The underlying disease, presence of emphysema, lesion size, lesion location, lesion depth, biopsy-related complications, and biopsy outcome were recorded. Lesion size was measured along the maximum long-axis diameter in the lung window setting. Lesion depth was measured from the pleura to the outer margin of the lesion along the needle path.

### CT-guided biopsy procedures

Informed consent was obtained from all patients before biopsy procedures. In all patients, the platelet count exceeded 100,000/μL, and the prothrombin time and activated prothrombin time were within normal limits. One experienced radiologist (20 years of experience) performed all CT-guided lung biopsies using a 4-slice MDCT scanner (LightSpeed Plus; GE Healthcare, Milwaukee, WI, USA).

Contrast-enhanced chest CT was available for review in all patients. The procedures were performed with patients in a prone, supine, oblique, or lateral decubitus position to provide the shortest distance between the lesion and pleural surface. The actual biopsy route was determined after obtaining a short spiral scan of the region of interest with a row of radiopaque markers on the chest with the patient’s holding his/her breath after inspiration or expiration. In all patients, the biopsy was performed using non-coaxial technique using an automated biopsy gun with an 18-gauge cutting needle (Bard Magnum, Covington, GA, USA) with a penetration depth of 15 or 17 mm. After puncturing the skin, the patient was instructed to hold his/her breath and the pleural puncture was subsequently made. The position of the needle tip was confirmed by obtaining limited CT images around the lesion at 5-mm thickness.

When lesion targeting failed, the operator usually tried to reposition the needle. However, in selective cases, a second attempt was made using another needle through a different puncture site, with the first needle retained in position: (1) when the lesion location was inconsistent in several repeated scans because of poor cooperation or poor respiration holding capacity by the patient and (2) the puncture site had to be shifted more than 1 cm from the initial site. The first needle was retained in position during the procedure and removed after completing tissue sampling with the second needle.

After tissue sampling, all patients underwent immediate CT scanning to detect procedure-related complications. Patients were asked to lie on the puncture site for the first 4 h during which coughing was discouraged. Inspiration upright posteroanterior chest radiographs were taken at 4 and 24 h after the biopsy. Pneumothorax was considered to be delayed if it appeared on the chest PA after 4 h or later.

After completion of biopsy, the operator described details of the biopsy procedure with a drawing, radiologic impression, risk factors for biopsy, patient’s position, biopsy route, difficulties in the procedure, causes of the failed targeting if occurred, number of obtained core samples, number of biopsy needles used, and procedure-related complications. Biopsy results.

## Results

Of the 17 patients, there were 9 males and 8 females with a mean age of 67.2 years (range 40–84 years). The mean size of the pulmonary lesions was 25.4 mm (range 12–42 mm). The location of the lesions was the right upper lobe in 3, the right middle lobe in 1, the right lower lobe in 6, the left upper lobe in 4, and the left lower lobe in 3 patients. Six patients had emphysema that was diffuse or localized around the target lesion.

In all 17 patients, we successfully obtained core sample(s) using the second needle. One core sample was obtained in 15 patients and 2 in 2 patients. The biopsy diagnoses were malignant in 9 and benign in 8 patients. Histopathological results included adenocarcinoma (*n* = 6), squamous cell carcinoma (*n* = 3), tuberculosis (*n* = 3), organizing pneumonia (*n* = 1), and chronic inflammation (*n* = 3). In 1 patient, the biopsy specimen was inadequate for a pathological interpretation due to an insufficient amount, and the final diagnosis was pneumonia.

Regarding difficult factors in biopsy, all 17 patients were not fully cooperative and did not follow respiration instruction. In addition, most patients had other lesion associated factors that were summarized in Table 1.

**Table 1:** Difficult factor(s) in biopsy procedure

Causes	No. patients (n = 17)
Poor cooperation/poor breath holding capacity	17
Long biopsy path (lesion depth, >5 cm)	3
Small size (≤2 cm)	5
Flat lesion (lesion thickness, ≤1 cm)	2
Lesion covered by ribs or scapula	9
Pre-existing pneumothorax	1

**Fig. 1:**
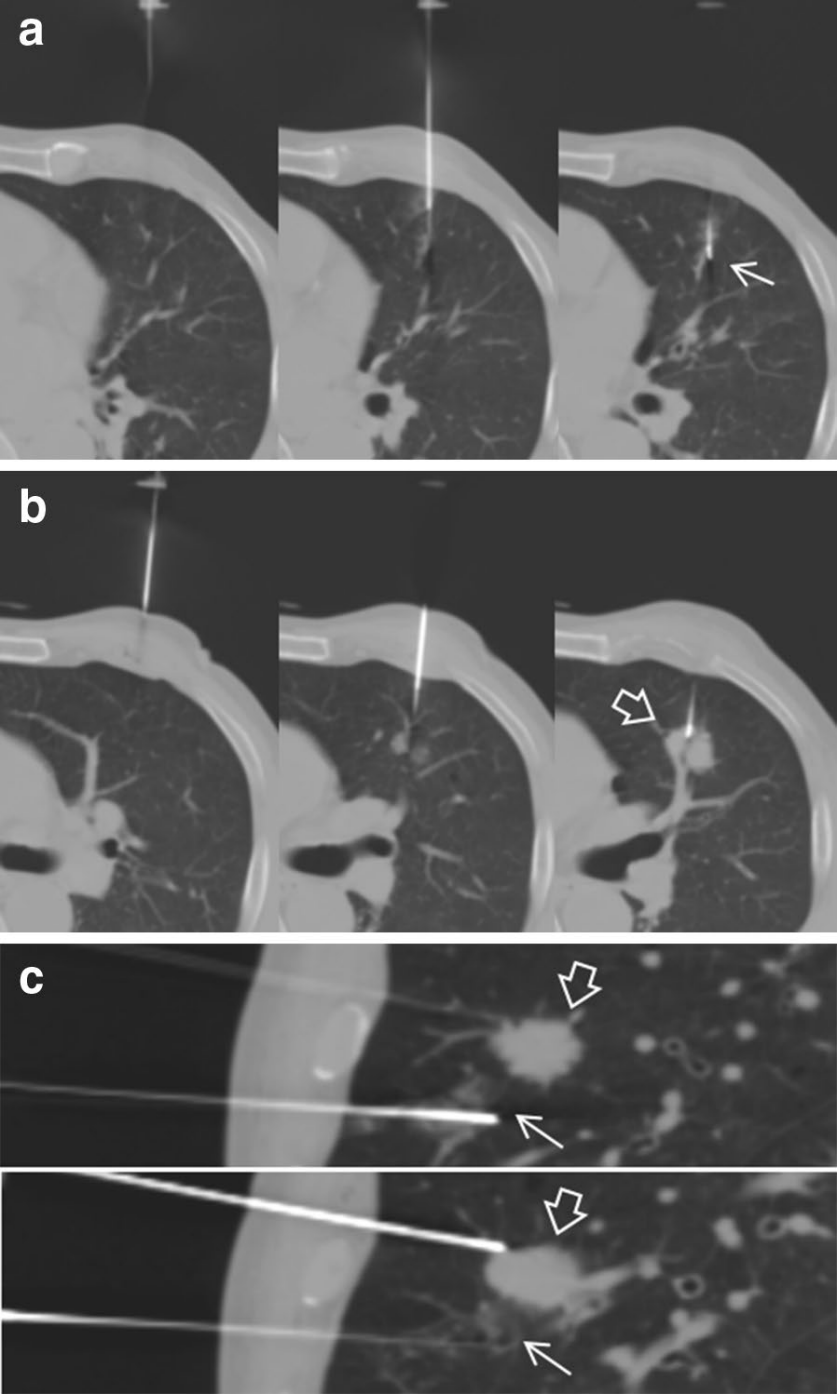
A 63-year-old man with a 2 cm sized nodule in left upper lobe (patient 8 in the Table 2). The biopsy proceeded in a supine position and a left 5th–6th intercostal approach. **a** In the first attempt, the needle was placed in the lung parenchyma (*arrow*) below the target. Because the patient did not reproduce the designated respiration, the operator modified the puncture site and needle direction based on the relationship between the first needle and target. **b** Leaving the malpositioned needle in position, the second needle was successfully inserted into the target nodule (*empty arrow*) above the first needle. **c** Sagittal reconstruction of the lesion and inserted needles. After the biopsy, there was no procedure-related complication. Histopathological examination showed a squamous cell carcinoma

**Fig. 2:**
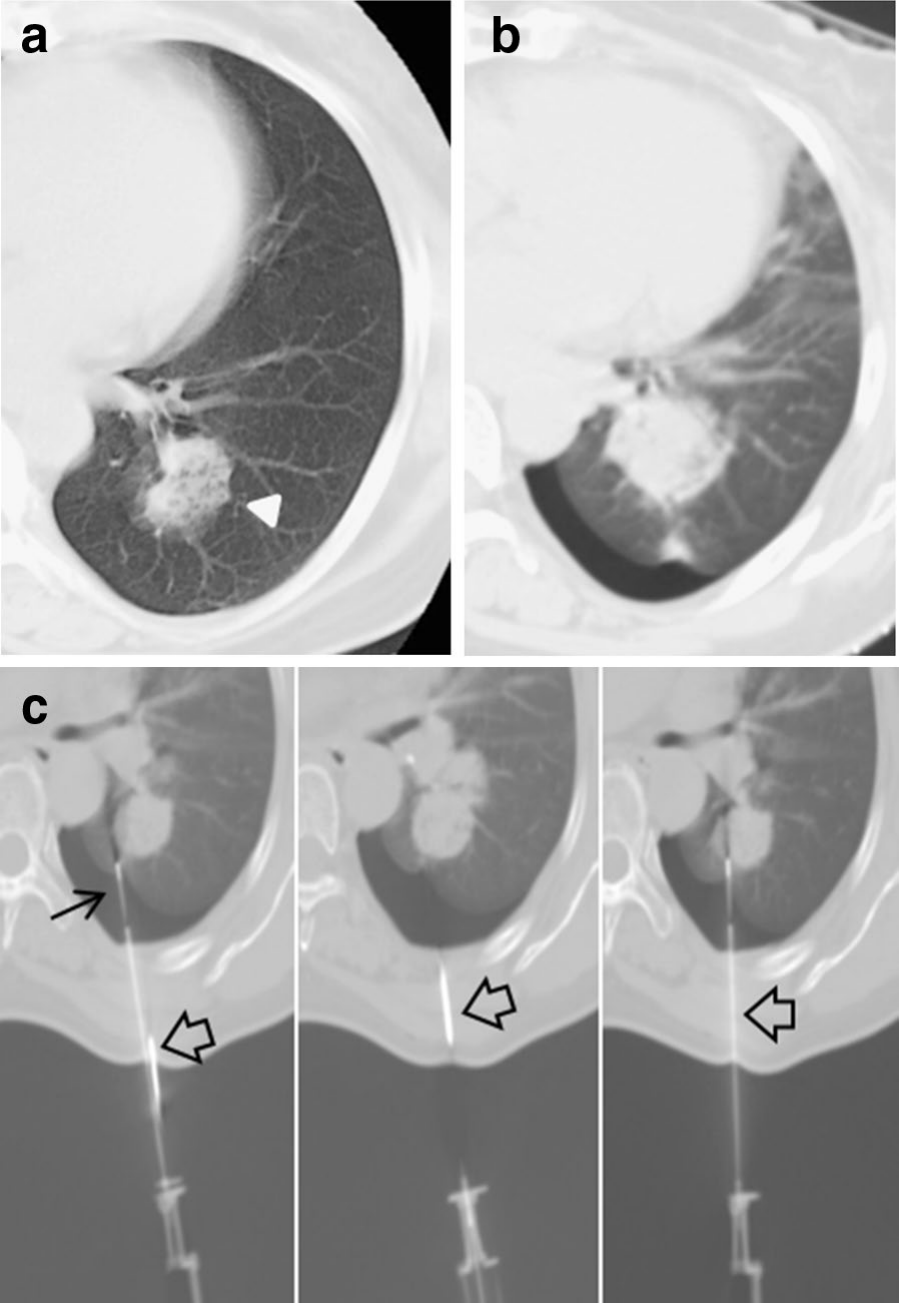
A 57-year-old woman with pre-biopsy pneumothorax (patient 7 in the Table 2). The patient underwent a transbronchial lung biopsy a day before, but insufficient tissue was obtained for a pathological diagnosis. **a** Axial unenhanced CT scan shows a 4.2 cm sized mass (*arrowhead*) in the left lower lobe superior segment. **b** Pneumothorax is present in a pre-biopsy CT scan. **c** Because of movement of the lung, the first needle was malpositioned in the lung parenchyma adjacent to the target (*thin arrow*). Instead of removing the malpositioned needle, a second attempt was made using another needle, expecting that the first needle could restrain the lung. The second needle successfully reached the target (*empty arrow*). A histopathological examination of biopsy specimen revealed an adenocarcinoma

The malpositioned first needle in the lung parenchyma served as a reference point for targeting the lesion in all 17 patients, thus playing a role in navigation for the second attempt. When the first biopsy attempt, based on marked puncture site and initially estimated needle direction, failed to target the lesion and the patient did not reproduce the designated respiration, the operator calculated the relative location and direction of the target to the location of the malpositioned needle tip and retried lesion targeting using another needle (Fig. 1). Another use of the malpositioned needle was as an anchor to restrict the movement of the lung, as seen in 4 patients. The malpositioned needle played a role in limiting lung movement in specific situations of increased lung mobility such as pneumothorax, or large respiratory movement due to poor respiration holding capacity (Fig. 2).

Postprocedure pneumothorax that developed in 4 patients (23.5 %) was detected through post-procedure CT scans in 2 patients and through follow-up chest radiographs taken at 4 or 24 h in 2 patients. The patient who already had pneumothorax was not included because the degree of pneumothorax did not increase after the procedure. None of the patients required chest tube insertion. Parenchymal hemorrhage developed in 3 (17.6 %) patients; however, it was resolved spontaneously without any treatment.

Patients’ demographics, the use of the first needle, biopsy results, and procedure-related complications were summarized in Table 2.

**Table 2:** Characteristics of patients who underwent CT-guided biopsy of lung lesions using two needles

Pt	Sex/age	Size (cm)	Location	Underlying disease	Difficulties for biopsy (other than poor cooperation)	Use of malpositioned needle	Biopsy results	Complication
1	F/78	2.5	RUL, posterior	AML		Navigation	Organizing pneumonia	No
2	M/61	2.2	RUL, posterior	CVA		Navigation	Tuberculosis	Parenchymal hemorrhage
3	M/70	2.4	RLL, anterior basal	CVA	Poor breath holding capacity, deep location^a^ (5 cm)	Navigation, anchor^c^	Adenocarcinoma	No
4	M/63	1.8	RLL, superior		Small lesion size^b^, covered by ribs, poor breath holding capacity	Navigation, anchor	Tuberculosis	Pneumothorax
5	F/70	2.8	RLL, lateral basal	Emphysema	Covered by ribs	Navigation	SCC	No
6	F/84	3.6	LUL, posterior	CVA	Deep location (8 cm)	Navigation	Adenocarcinoma	No
7	F/57	4.2	LLL, superior		Pneumothorax	Navigation, anchor	Adenocarcinoma	Parenchymal hemorrhage
8	M/63	2	LUL, anterior	Emphysema	Small lesion size, covered by ribs	Navigation	SCC	No
9	M/77	3.4	RML, lateral	Emphysema	Covered by ribs and scapula	Navigation	Chronic inflammation	Delayed pneumothorax^d^
10	F/57	3.5	RLL, superior		Flat lesion, covered by ribs	Navigation	Adenocarcinoma	Delayed pneumothorax
11	M/75	1.2	LUL anterior	Emphysema	Poor breath holding capacity, small lesion size	Navigation, anchor	Adenocarcinoma	No
12	M/40	2.3	LUL, anterior		Covered by ribs (subcostal location)	Navigation	Chronic inflammation	No
13	F/66	3	RUL posterior	AML	Flat lesion, deep location (5 cm), covered by ribs and scapula	Navigation	Chronic inflammation	No
14	M/67	3.5	LLL, posterior basal		Covered by ribs	Navigation	Tuberculosis	Pneumothorax
15	F/70	2	LLL, posterior basal		Small lesion size	Navigation	Adenocarcinoma	No
16	F/74	2.8	RLL, anterior basal	Emphysema	Covered by ribs	Navigation	SCC	Parenchymal hemorrhage
17	M/70	1.2	RLL, posterior basal	Emphysema	Small lesion size	Navigation	Non-diagnostic	No

*Pt* patient, *RUL* right upper lobe, *RLL* right lower lobe, *RML* right middle lobe, *LUL* left upper lobe, *LLL* left lower lobe, *AML* acute myelocytic leukemia, *CVA* cerebrovascular accident, *SCC* Squamous cell carcinoma

^a^Lesion depth, >5 cm

^b^Lesion size, ≤2 cm

^c^Anchoring the lung

^d^Pneumothorax occurred 4 h or later after the procedure

## Discussion

Overall diagnostic yields of CT-guided lung biopsy are excellent but somewhat variable depending on the degree of difficulty of the attempted procedure and the operator’s experience (Winokur et al. 2013; Montaudon et al. 2004). In the era of MDCT, as more indeterminate lesions are detected in chest CTs, the thoracic radiologist often encounters patients with difficult lesions or who are poorly cooperative for percutaneous biopsies. For success in CT-guided biopsies, patient cooperation is indispensable (Moore 1998). In standard CT-guided biopsies without the use of real-time monitoring tools, the final needle path in the lung is blind and unstable breath holding during the procedure or any movement would render the initial localization of the lesion inaccurate, causing the needle to be inserted outside the target. The risk of targeting failure would be higher when a small lesion is located in more movable areas of the lungs, such the lower lobes or anterior segment (Hiraki et al. 2009; Takeshita et al. 2015).

CT fluoroscopy can be helpful in biopsies of difficult cases because it enables visualization of the lesion and needle manipulation in real time (Gianfelice et al. 2000; Kim et al. 2011). Although CT fluoroscopy is a promising tool, it is not yet available to many radiologists. In addition, there is additional radiation exposure to both the operator and patient during procedures and some limitations to needle handling within the CT gantry (Kim et al. 2011; Pereira et al. 2011; Prosch et al. 2012).

When the lesion is covered by the ribs or scapula, the operator cannot target the lesion in a vertical direction. In 9 of the 17 patients, biopsies were performed using an oblique approach in the craniocaudal or mediolateral direction. This is technically more difficult than a vertical approach and known to increase the rate of pneumothorax because oblique pleural puncture angle creates elongation of the pleural hole (Ko et al. 2001; Li et al. 2013). Multiplanar reconstruction or gantry tilt can be helpful for the procedure (Kimura et al. 2004; Yueh et al. 1989); however, these steps take extra time and tissue sampling would like wise be unsuccessful if the patient’s respiration is inconsistent.

When a biopsy needle is misplaced in the normal lung parenchyma distinct from the target, the operator usually removes the needle and re-attempts targeting. However, in select patients, instead of removing a malpositioned needle, we re-attempted lesion targeting using a second needle through another puncture site while the first needle was in position. The malpositioned needle retained in lung parenchyma was beneficial in performing the second attempt. First, when the patient did not reproduce designated respiration and re-targeting could not depend on CT images, the operator determined the lesion location based on the location of the malpositioned needle. Although the patient’s respiration was irregular, the relative position between the lesion and inserted needle did not change; thus, the operator calculated the distance of the lesion relative to the malpositioned needle and determined a new puncture site and needle direction. Second, the malpositioned needle acted as an anchor to hold the lung. In a patient with pre-existing pneumothorax, the first needle failed to target the lesion because of the movability of the lung. In the second trial using another needle, the malpositioned needle held the lung in place, which was helpful for accurate lesion targeting by the second needle. When the patient’s respiratory movement was large and variable, the inserted needle restricted the patient’s respiratory motion to a limited range, enabling the operator to estimate the lesion location. Furthermore, the operator could control the patient’s respiratory movement by observing the needle movement. Because pneumothorax did not occur or progress while the malpositioned needle was ‘holding’ the lung, the operator could proceed with another biopsy attempt.

In the present study, pneumothorax occurred in 4 of 17 patients (23.5 %). Since the incidence of pneumothorax in patients undergoing transthoracic lung biopsies is reportedly between 9–59.6 %, the pneumothorax rate in our study was within the acceptable range (Nakamura et al. 2011; Priola et al. 2010; Halloush et al. 2007; Saji et al. 2002; Boskovic et al. 2014; Khan et al. 2008). Despite controversy, repeated pleural punctures are a known risk factor of biopsy related pneumothorax (Kakizawa et al. 2010; Nour-Eldin et al. 2015). The low pneumothorax and chest tube placement rates in this study could be related to the strict pneumothorax precautions, including puncture side-down positioning for 4 h and discouraging coughing (Moore et al. 1990).

In the present study, despite many difficulties and poor patient cooperation, we conducted CT-guided biopsies without real time monitoring tools to make a diagnosis and determine treatment plans. There were several limitations in our study. First, the study population was small. However, it is difficult to implement this 2-needle technique in a large population because the first needle was inevitably malpositioned in the normal lung parenchyma. Second, the study was retrospective and the results from using the two needle technique and from simple reinsertion of a single needle were not compared. Third, there was possible patient selection and interpretation bias because all procedures were performed by one experienced radiologist. However, radiologists who have different levels of experience or prefer to use co-axial technique may be able to try this method when the first targeting fails because of patient associated factors.

## Conclusion

Conventional CT-guided percutaneous lung biopsy using two needles could be tried in select patients after consideration of the risks and benefits of the procedure. The malpositioned needle retained in the lung parenchyma could serve as a navigation point estimating the lesion location and an anchor of the lung.

## Abbreviations

CT: computed tomography
MDCT: multidetector computed tomography
